# Effect of a 12‐week endurance training program on force transfer and membrane integrity proteins in lean, obese, and type 2 diabetic subjects

**DOI:** 10.14814/phy2.14429

**Published:** 2020-05-01

**Authors:** Sebastian Jannas‐Vela, Henning T. Langer, Hugo Marambio, Keith Baar, Hermann Zbinden‐Foncea

**Affiliations:** ^1^ Exercise Physiology Laboratory School of Kinesiology Universidad Finis Terrae Santiago Chile; ^2^ Department of Neurobiology, Physiology and Behavior University of California Davis CA USA; ^3^ Centro de Salud Deportiva Clinica Santa Maria Santiago Chile

**Keywords:** dystrophin, endurance exercise, force transfer proteins, obese, type II diabetes

## Abstract

The mechanisms accounting for the loss of muscle function with obesity and type 2 diabetes are likely the result of a combination of neural and muscular factors. One muscular factor that is important, yet has received little attention, is the protein machinery involved in longitudinal and lateral force transmission. The purpose of this study was to compare the levels of force transfer and membrane integrity proteins before and after a 12‐week endurance training program in lean, obese, and obese type 2 diabetic adults. Nineteen sedentary subjects (male = 8 and female = 11) were divided into three groups: Lean (*n* = 7; 50.3 ± 4.1 y; 69.1 ± 7.2 kg); Obese (*n* = 6; 49.8 ± 4.1 y; 92.9 ± 19.5 kg); and Obese with type 2 diabetes (*n* = 6; 51.5 ± 7.9 years; 88.9 ± 15.1 kg). Participants trained 150 min/week between 55% and 75% of VO_2max_ for 12 weeks. Skeletal muscle biopsies were taken before and after the training intervention. Baseline dystrophin and muscle LIM protein levels were higher (~50% *p* < .01) in lean compared to obese and type 2 diabetic adults, while the protein levels of the remaining force transfer and membrane integrity proteins were similar between groups. After training, obese individuals decreased (−53%; *p* < .01) the levels of the muscle ankyrin repeat protein and lean individuals decreased dystrophin levels (−45%; *p* = .01), while the levels of the remaining force transfer and membrane integrity proteins were not affected by training. These results suggest that there are modest changes to force transfer and membrane integrity proteins in middle‐aged individuals as a result of 12 weeks of lifestyle and training interventions.

## Graphical abstract

A 12‐week endurance training regimen has a relatively small effect on the levels of force transfer and membrane integrity proteins of lean, obese and type 2 diabetic individuals. Only dystrophin and muscle ankyrin repeat protein were decreased after training in lean and obese individuals, respectively.

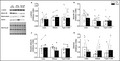

## 1 INTRODUCTION

Obesity affects approximately 650 million people, accounting for 13% of the world's adult population (World Health Organization, 2016). It is caused by an energy imbalance between calories consumed and calories expended, mainly as a result of increased intake of energy‐dense foods and decreased physical activity. Obesity is linked to many physiological conditions, of which type 2 diabetes is one of the most common (Eckel et al., 2011; Leitner et al., 2017). Obesity and type 2 diabetes have been suggested to accelerate the loss of muscle strength with age (Park et al., 2006) and muscle strength has been acknowledged as the critical factor for determining physical disability and mortality (Manini & Clark, 2012).

The mechanisms accounting for muscle strength loss have been attributed to a combination of neural and muscular factors (Manini & Clark, 2012). One muscular factor that is important, yet has received little attention, is the protein machinery involved in longitudinal and lateral force transmission. Force is transmitted both longitudinally, through the Z‐lines of sarcomeres in series to the myotendinous junction and on to the tendon, and laterally, from the sarcomeres through the surrounding basal lamina and extracellular matrix (ECM) to the tendon (Hughes, Wallace, & Baar, 2015). The longitudinal force transmission system comprises proteins that are within the thick and thin filaments, the Z‐lines (α‐actinin) and the proteins that anchor the thick (ankyrin repeat proteins) and thin (muscle LIM proteins) filaments (Hughes et al., 2015). Meanwhile, the lateral force transmission pathway is composed of costameric proteins and intermediate filaments (Henderson, Gomez, Novak, Mi‐Mi, & Gregorio, 2017).

Costameres are protein complexes that connect the cytoskeletal system within a fiber to the overlying ECM (Pardo, Siliciano, & Craig, 1983). One costameric component is the dystrophin‐associated glycoprotein complex (DGC), comprised of dystrophin, dystroglycan, and sarcoglycan proteins. During contraction, the DGC links the sarcomeric proteins of adjacent sarcomeres by forming a functional connection through the sarcolemma to the ECM (Lovering & De Deyne, 2004; Ramaswamy et al., 2011). This connection functions like rivets to prevent shearing between adjacent fibers (Claflin & Brooks, 2008) and thus protects individual fibers from contraction‐induced injury. Costameres also include integrin proteins that bind intermediate filaments (IFs). The IFs link the contractile proteins, and also organelles such as mitochondria and nuclei, to costameres thereby providing cellular integrity, force transmission, and a potential source of mechanochemical sensing (Capetanaki, Bloch, Kouloumenta, Mavroidis, & Psarras, 2007). The IF network is composed of proteins that are grouped into six types, with desmin being the main IF expressed in skeletal muscle (Goldfarb, Olivé, Vicart, & Goebel, 2008). Thus, desmin is considered a significant contributor to the maintenance of skeletal muscle structure, function, and force transmission.

In response to contraction‐induced injury, two proteins, dysferlin and annexin, have been purported to play a key role in the maintenance of membrane integrity. Upon mechanical stress, dysferlin and annexin accumulate at lesion sites within the sarcolemma, forming a membrane patch to contain the damage and prevent calcium influx (Han & Campbell, 2007; Lennon et al., 2003). Therefore, an elevation of dysferlin and annexin levels can be indicative of membrane disruption/damage and subsequent repair (Han & Campbell, 2007).

It is well documented that endurance exercise improves metabolic capacity of skeletal muscle (Holloszy & Coyle, 1984; Ingjer, 1979); however, there is limited research on the role of endurance exercise on skeletal muscle force transfer and membrane integrity proteins. Furthermore, whether these proteins are expressed differently in middle‐aged healthy, obese, and type 2 diabetic adults has yet to be determined. This is of major importance as the force transfer and membrane repair apparatus are integral for the maintenance of skeletal muscle function in diseased populations. Thus, the purpose of this study was to compare the levels of force transfer and membrane integrity proteins before and after a 12‐week endurance training program in lean, obese, and type 2 diabetic adults.

## 2 METHODS

The study was approved by the Universidad Finis Terrae Research Ethics Board and conformed to the standards set by the Declaration of Helsinki. This study used a portion of data collected from individuals participating in an intervention examining the effects of endurance exercise training on insulin sensitivity and skeletal muscle inflammation. Briefly, 19 sedentary subjects (male = 8 and female = 11) who maintained a stable body weight 6 months prior to study were divided into three groups based on body weight and blood glucose control: Lean (50.3 ± 4.1 years; 69.1 ± 7.2 kg); Obese (49.8 ± 4.1 years; 92.9 ± 19.5 kg); and Obese with type 2 diabetes (T2D) (51.5 ± 7.9 years; 88.9 ± 15.1 kg).

The inclusion criteria for Lean and Obese groups were having a BMI between 18 and 25 kg/m^2^ and between 30 and 40 kg/m^2^, respectively. Neither group had a family history of T2D. In the case of T2D group, the inclusion criteria was having a BMI between 30 and 40 kg/m^2^ and having been diagnosed with diabetes mellitus type 2, defined as fasting glycaemia over 126 mg/dl or over 200 mg/dl, 2 hr following a 75 g glucose challenge. The exclusion criteria for the study were as follows: physical limitations that would make it difficult to complete the exercise program, uncontrolled hypertension (blood pressure > 160/90 mmHg), history of heart disease, myocardial infarction, stroke, and any other contraindications to exercise. Written informed consent was received from each subject following a detailed explanation of the experimental protocol and any associated risks. Subjects were instructed to maintain consistent dietary and exercise habits, outside of the intervention, throughout the study.

### 2.1 Study design

Participants were assigned to one of three groups according to their anthropometric and metabolic data: lean (*n* = 7); obese (*n* = 6); and type II diabetic (T2D) (*n* = 6). At least 2 days before the training intervention, participants underwent an incremental cycling test to determine maximal oxygen consumption (VO_2_max). The participants then trained for 12 weeks at the laboratory according to guidelines from World Health Organization (2010): 150 min/week between 55% and 75% of VO_2_max. Skeletal muscle biopsies were taken before and after the 12‐week training intervention.

### 2.2 Incremental cycling test

The incremental cycling test was performed on a cycle ergometer (Matrix Fitness System). The test started with participants pedaling at 50 W for 4 min, followed by an increase of 25 W per minute for female participants and 50 W for male participants, until voluntary exhaustion. Cadence was kept at 60 revolutions per minute and participants were verbally encouraged to perform their maximum effort during the test. Exhaled gases (VO_2_ and VCO_2_) were analyzed using an open‐circuit gas analyzer (Medisoft).

### 2.3 Training

Participants performed the training intervention on a cycle ergometer (Matrix Fitness System) 3 times per week for 12 weeks. During the first six sessions training duration was increased from 20 to 50 min at 55% of VO_2_max. The following 30 sessions were performed for 50 min at 65%–75% of VO_2_max. The heart rate observed during 55%–75% of VO_2_max test was used as training reference.

### 2.4 Muscle biopsies

Muscle biopsies (total ~80–150 mg) were obtained under local anesthesia (2% lidocaine without epinephrine) from the vastus lateralis muscle, using the percutaneous needle biopsy technique described by Bergström (1975). A portion (~30 mg) of muscle frozen in liquid nitrogen and was used for biochemical analyses.

### 2.5 Western blotting

Frozen muscles were powdered and homogenized in sucrose lysis buffer (50 mM Tris pH 7.5, 250 mM sucrose, 1 mM ethylenediaminetetraacetic acid, 1 mM ethylene glycol‐bis(beta‐aminoethyl ether)‐N,N,N′,N′‐tetraacetic acid, 1% Triton X 100, and protease inhibitors). The supernatant was collected following centrifugation at 10,000*g* for 5 min and protein concentrations were determined in duplicate using the DC protein assay (Bio‐Rad). Twenty‐four micrograms of protein was subjected to SDS‐PAGE on 4%–20% Criterion TGX stain‐free protein gels (Bio‐Rad) and transferred to nitrocellulose membrane for 1 hr. Membranes were blocked in 1% fish skin gelatin dissolved in Tris‐buffered saline with 0.1% Tween‐20 for 1 hr and then probed with primary antibody overnight at 4°C. The next day, membranes were washed and incubated with horseradish peroxidase‐conjugated secondary antibodies at 1:10,000 for 1 hr at room temperature. Immobilon Western Chemiluminescent horseradish peroxidase substrate (Millipore) was then applied to the membranes for protein band visualization by chemiluminescence. Image acquisition and band quantification were performed using the ChemiDoc™ MP System and Image Lab 5.0 software (Bio‐Rad). Total protein within each lane, as determined by the fluorescent signal obtained following 1‐min UV activation, was used as the normalization control for all blots.

The following commercially available antibodies were used: dystrophin (Santa Cruz, Cat no. 365954), β‐dystroglycan (Hybridoma Bank, Cat no. MANDAG2), α‐sarcoglycan (Hybridoma Bank Cat no. IVD3 A9), laminin‐2α (Santa Cruz, Cat no. 20142), desmin (Santa Cruz, Cat no. 271677), α‐actinin (Santa Cruz, Cat no. 17829), muscle ankyrin repeat protein (MARP) (Santa Cruz, Cat no. 138111), and muscle LIN‐11, ISL‐1, and MEC‐3 domain (LIM) protein (Santa Cruz, Cat no. 166930). Both syntrophin and sarcospan cross‐reacted with the antibody for dystrophin (Cat no. 365954) and were determined by molecular weight.

### 2.6 Statistical analysis

All data are presented as means ± standard error of the mean (*SEM*). A two‐way repeated‐measures ANOVA with a post hoc Fisher's LSD test was used to examine changes in protein content using group and time as fixed effects. Statistical significance was declared at the .05 level. GraphPad Prism program, version 7.0 (GraphPad Software, Inc.), was used for statistical analysis. All data were checked for normality before any analyses were performed.

## 3 RESULTS

### 3.1 Aerobic capacity

After 12 weeks of moderate‐intensity endurance training, there was a significant increase (*p* < .01) in VO_2_max (ml/min/kg) in all intervention groups (Lean: 23.0 ± 9.4 to 28.9 ± 8.8; Obese: 21.6 ± 5.8 to 28.0 ± 5.7; and T2D: 27.6 ± 9.3 to 32.0 ± 9.2).

### 3.2 Longitudinal force transfer proteins and desmin

**Figure 1:**
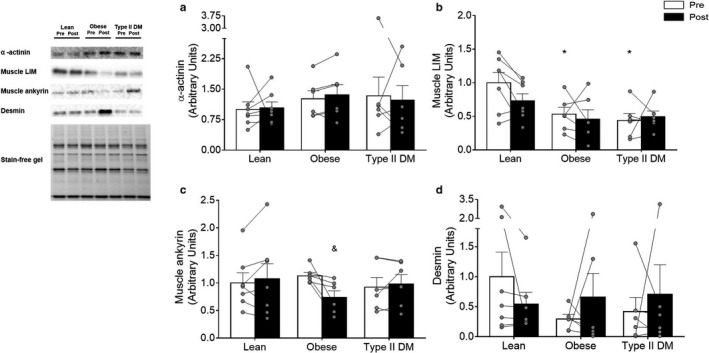
Longitudinal force transfer and desmin protein levels in lean, obese, and type 2 diabetic adults before (Pre – white bars) and after (Post – black bars) 12 weeks of endurance training. (a) α‐actinin; (b) muscle LIM; (c) muscle ankyrin; and (d) desmin protein levels in lean, obese, and type 2 diabetic adults. Values are reported as means ± *SEM*. *Significantly different from Lean pre; ^&^Significantly different from Obese Pre

Baseline protein content for desmin and the longitudinal force transfer proteins α‐actinin and MARP were similar between groups, whereas muscle LIM protein was higher (*p* < .01) in lean compared to obese (−47%) and type 2 diabetic (−56%) adults (Figure 1). After the 12‐week training period, obese individuals decreased (−53%; *p* < .01) the levels of MARP (Figure 1c). The levels of the other longitudinal force transfer proteins were not affected by training.

### 3.3 Lateral force transfer proteins

**Figure 2:**
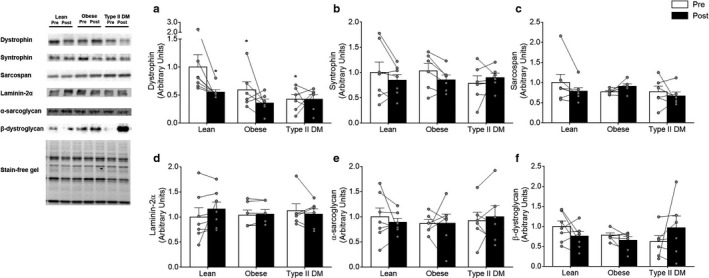
Lateral force transfer protein levels in lean, obese, and type 2 diabetic adults before (Pre – white bars) and after (Post – black bars) 12 weeks of endurance training. (a) Dystrophin; (b) syntrophin; (c) sarcospan; (d) laminin‐2α; (e) α‐sarcoglycan; and (f) β‐dystroglycan protein levels in lean, obese, and type 2 diabetic adults. Values are reported as means ± *SEM*. *Significantly different from Lean Pre

Baseline dystrophin levels were higher (*p* < .01) in lean compared with both obese (−41%) and type 2 diabetic (−58%) adults (Figure 2a). The other proteins within the dytrophin‐associated glycoprotein complex were similar between groups at baseline. In lean individuals, dystrophin protein decreased (−45%; *p* = .01) with training, whereas levels of the remaining lateral force transfer proteins were not affected by training in any of the intervention groups (Figure 2).

### 3.4 Membrane repair proteins

**Figure 3:**
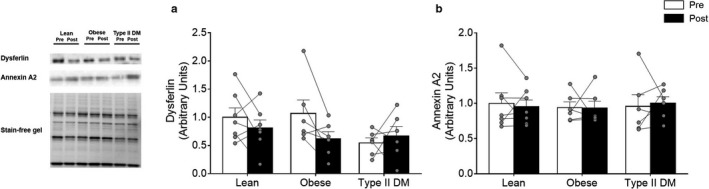
Membrane integrity protein levels in lean, obese, and type 2 diabetic adults before (Pre – white bars) and after (Post – black bars) 12 weeks of endurance training. (a) Dysferlin and (b) annexin A2 protein levels in lean, obese, and type 2 diabetic adults. Values are reported as means ± *SEM*. There were no differences within and between groups

The baseline levels of the membrane repair proteins dysferlin and annexin A2 were similar between groups and neither was significantly altered by training (Figure 3).

## 4 DISCUSSION

The major findings of this study were as follows: (1) that dystrophin and muscle LIM protein levels were higher in lean controls compared with obese and type 2 diabetic adults and (2) that a 12‐week endurance training program had a small effect on the levels of force transfer and membrane integrity proteins. Only the muscle ankyrin repeat protein (MARP) and dystrophin levels were altered by training in obese and lean individuals, respectively.

Obese and type 2 diabetic adults have been reported to have decreased muscle strength and increased propensity to muscle injury compared to their lean counterparts (Eckel et al., 2011; Kim & So, 2018). In this context we aimed to determine whether the levels of force transfer and membrane integrity proteins were different among lean, obese, and type 2 diabetic individuals. In support of the hypothesis that obesity and/or diabetes alters force transfer in muscle, the levels of dystrophin and muscle LIM proteins were higher in skeletal muscle of lean compared to obese and type 2 diabetics adults; while the levels of the other force transfer and membrane integrity proteins were similar between groups. Dystrophin links the cytoskeletal and motor proteins of the sarcomere to the sarcolemma and the overlying ECM (Gao & McNally, 2015). As a result, decreased levels of dystrophin are accompanied by significant reductions in lateral transmission of force and increased propensity for contraction‐induced muscle injury (Lovering & De Deyne, 2004; Ramaswamy et al., 2011). Similarly, muscle LIM protein provides a structural connection between α‐actinin and titin (Schallus, Fehér, Ulrich, Stier, & Muhle‐Goll, 2009), playing an important role in longitudinal force transfer. Therefore, in our study the increased levels of dystrophin and muscle LIM proteins in lean adults suggest that at baseline these subjects have better force transfer in both the lateral and longitudinal directions. Furthermore, the lower level of dystrophin protein may make muscle from obese and type 2 diabetic individuals more prone to contraction‐induced muscle injury (Hughes et al., 2015; Ramaswamy et al., 2011). In support of the current findings, Taub and colleagues have previously shown that muscles from diabetics and heart failure patients have very low levels of dystrophin protein (Taub et al., 2013). In contrast to the current work, Taub found that all of the lateral force transfer proteins were reduced in their patients. The likely explanation for the difference between the two studies is that the subjects in the Taub study also had heart failure and were significantly sicker to those in the current work. These data suggest that dystrophin protein loss may precede the loss of other force transfer proteins as a result of disease.

After 12 weeks of moderate‐intensity endurance training, we observed an unexpected decrease in the level of dystrophin protein in lean individuals. In fact, following training dystrophin levels in the lean subjects were similar to the levels found in obese and type 2 diabetics before training. This result was surprising, as previous studies have reported a trend for increased dystrophin after resistance exercise training in old (60–75 years) men (Kosek & Bamman, 2008), and unchanged levels in dystrophin protein after either resistance exercise training (Kosek & Bamman, 2008; Woolstenhulme, Conlee, Drummond, Stites, & Parcell, 2006) or endurance training (Parcell, Woolstenhulme, & Sawyer, 2009) in young men (<30 y). The discrepancy between the existing literature and the current work may be explained by the age of the participants, their starting fitness, or the intensity of the training interventions. It is possible that in the current subjects the moderate‐intensity endurance exercise resulted in hypertrophy of the quadriceps muscle (Harber et al., 2009; Hudelmaier et al., 2010; Konopka & Harber, 2014) and the additional contractile and regulatory proteins within the fiber resulted in the relative decrease in dystrophin protein that we detected by western blot. Furthermore, recent research suggests that changes in muscle hypertrophy after short‐term training are attributed to increases in cytoplasmic rather than myofibrillar protein levels (Haun et al., 2019). Therefore, it is likely that the lower levels of dystrophin observed after training in healthy adults (~45%) were due to sarcoplasmic expansion. In support of the current findings, a recent study showed that muscle dystrophin protein levels were significantly lower (~43%) in trained than untrained individuals (Vann et al., 2020). Either way, the low levels of dystrophin observed in obese and type 2 diabetic individuals before training is consistent with a growing literature showing that dystrophin protein is lost in metabolic and inflammatory disease (Gutierrez‐Salmean et al., 2014; Taub et al., 2013) and that this might play a role in the incidence of muscle injury in these populations (Hughes et al., 2015). Another potential message from the current work is the importance of prescribing resistance‐type exercise in all populations as a way to counteract the potential loss of dystrophin. Future studies are clearly warranted to elucidate the levels of force transfer and membrane integrity proteins in different populations and the adaptation of these proteins to different training modalities.

Along with dystrophin, MARP was decreased after endurance training in obese individuals. MARPs are a family of proteins located within the I‐band of the sarcomere where they bind to the N2A region of titin as well as to the nebulin anchoring protein myopalladin (Kojic, Radojkovic, & Faulkner, 2011). MARP knockout mice show small changes in resting sarcomere length and greater loss in torque in the acute period after eccentric contractions, indicating greater contraction‐induced injury (Barash et al., 2007). However, there are no functional impairments at later recovery periods, suggesting that other members of the family, such as the cardiac ankyrin repeat, may be able to compensate for the loss in MARP (Hughes et al., 2015). Thus, the observed decrease in MARP after training may not be sufficient to negatively affect muscle architecture or function in the obese subjects.

In conclusion, this study reported minor differences among lean, obese, and type 2 diabetic adults in the levels of dystrophin and muscle LIM protein level. Both dystrophin and muscle LIM were higher in lean compared to obese and type 2 diabetic adults. The lower dystrophin levels may play a role in the elevated muscle damage seen following eccentric exercise (Kim & So, 2018). Twelve weeks of endurance training increased the aerobic capacity in all groups; however, training had a relatively small effect on the levels of force transfer and membrane integrity proteins, as only dystrophin and muscle ankyrin repeat protein were decreased after training in lean and obese individuals, respectively. These results suggest that the levels of skeletal muscle force transfer and membrane integrity proteins are in general resistant to moderate changes in lifestyle and training.

## CONFLICT OF INTEREST

The authors declare no conflict of interest.
